# Rho Kinase Inhibitors as a Neuroprotective Pharmacological Intervention for the Treatment of Glaucoma

**DOI:** 10.7759/cureus.28445

**Published:** 2022-08-26

**Authors:** Nisha M Thomas, Prachi Nagrale

**Affiliations:** 1 Ophthalmology, Jawaharlal Nehru Medical College, Datta Meghe Institute of Medical Sciences, Wardha, IND

**Keywords:** retinal ganglion cell, intraocular pressure, axon regeneration, neuroprotection, rho kinase inhibitors, glaucoma

## Abstract

Glaucoma is a leading cause of irreversible blindness, and its prevalence has led to research into treatment modalities for glaucoma to prevent the progression of the disease. The primary treatment for glaucoma that has been extensively used is ocular hypotensives to reduce raised intraocular pressure. This treatment has its drawbacks due to the existence of other variants of glaucoma, such as normal-tension glaucoma, where the intraocular pressure is measured to be within regular levels. Hence, there is a need for new treatment interventions which can deliver a better prognosis for glaucoma. Neuroprotection is a new concept studied recently, and neuroprotective agents are being developed for glaucoma therapy. Rho kinase inhibitors are one such neuroprotective agent, and the most recent addition to the class of ocular hypotensives, where they function by reducing raised intraocular pressure. Its neuroprotective capabilities, such as cell survival and axon regeneration, are yet to be determined in detail. This literature review article aims to look into the need for new treatments such as neuroprotection to prevent the progression of glaucoma and the efficacy of rho kinase inhibitors in the treatment of glaucoma, with particular emphasis on its neuroprotective abilities. It also aims to identify the limitations that can occur while approaching neuroprotective therapy, as well as how it can enable future treatment modalities. By exploring this field, blindness caused by progressive glaucoma can be halted and managed by glaucoma therapy.

## Introduction and background

Blindness in the world is caused by cataracts, glaucoma, refractive errors, diabetic retinopathy, and macular degeneration, among others. Glaucoma is the second most common leading cause of irreversible blindness in the world and is prevalent in about 66.8% of the population [1]. In India, around 12 million individuals are estimated to be suffering from glaucoma, of whom 1.5 million people have progressive blindness, making it the third most prevalent cause in the country [2]. More than 75% of glaucoma cases remain undetected, contributing to the iceberg phenomenon of the disease.

Description of disease

Glaucoma refers to a group of optic neuropathies responsible for optic nerve head excavation/cupping leading to retinal ganglion cell apoptosis. Raised intraocular pressure remains one of the highest risk factors for the cause and development of glaucoma [3]. Glaucoma is classified into two main categories: primary glaucoma and secondary glaucoma. Primary glaucoma is further divided into open-angle glaucoma (OAG) and closed-angle glaucoma (CAG), out of which OAG is seen as the more incidental type compared to CAG [4]. Intraocular pressure of more than 21 mmHg is a known and identifiable risk factor for the occurrence of OAG, as raised intraocular pressure can lead to mechanical impairment of the optic nerve and thereby destroy the retinal ganglion cells [5]. The main approach for therapeutic modalities is focused on trabecular meshwork and aqueous humor outflow pathways [6]. Treatment interventions for the same include topical use of anti-glaucoma drugs, laser surgery, and minimally invasive glaucoma surgery to reduce intraocular pressure [7]. It has been found that the risk of blindness among patients treated for OAG is high and estimated to be up to 27% [8].

Another category of glaucoma exists called normal-tension glaucoma (NTG), wherein the intraocular tension is below the standard range, i.e., less than 21 mmHg. Despite being within the normal range of intraocular pressure, some patients develop progressive degeneration of retinal ganglion cells. A study conducted in Japan found that only 8% of patients presented with OAG, whereas 48% of patients showed NTG [9]. Another study conducted in Singapore showed that around 50% of the population presented with NTG [10]. Hence, it can be concluded that other unidentified intraocular pressure-independent risk factors also play a role in causing retinal ganglion cell death. This raises the question of whether reducing intraocular pressure is not the sole therapeutic approach that can be adopted to prevent disease progression.

## Review

Mechanism of retinal ganglion cell death

**Figure 1:**
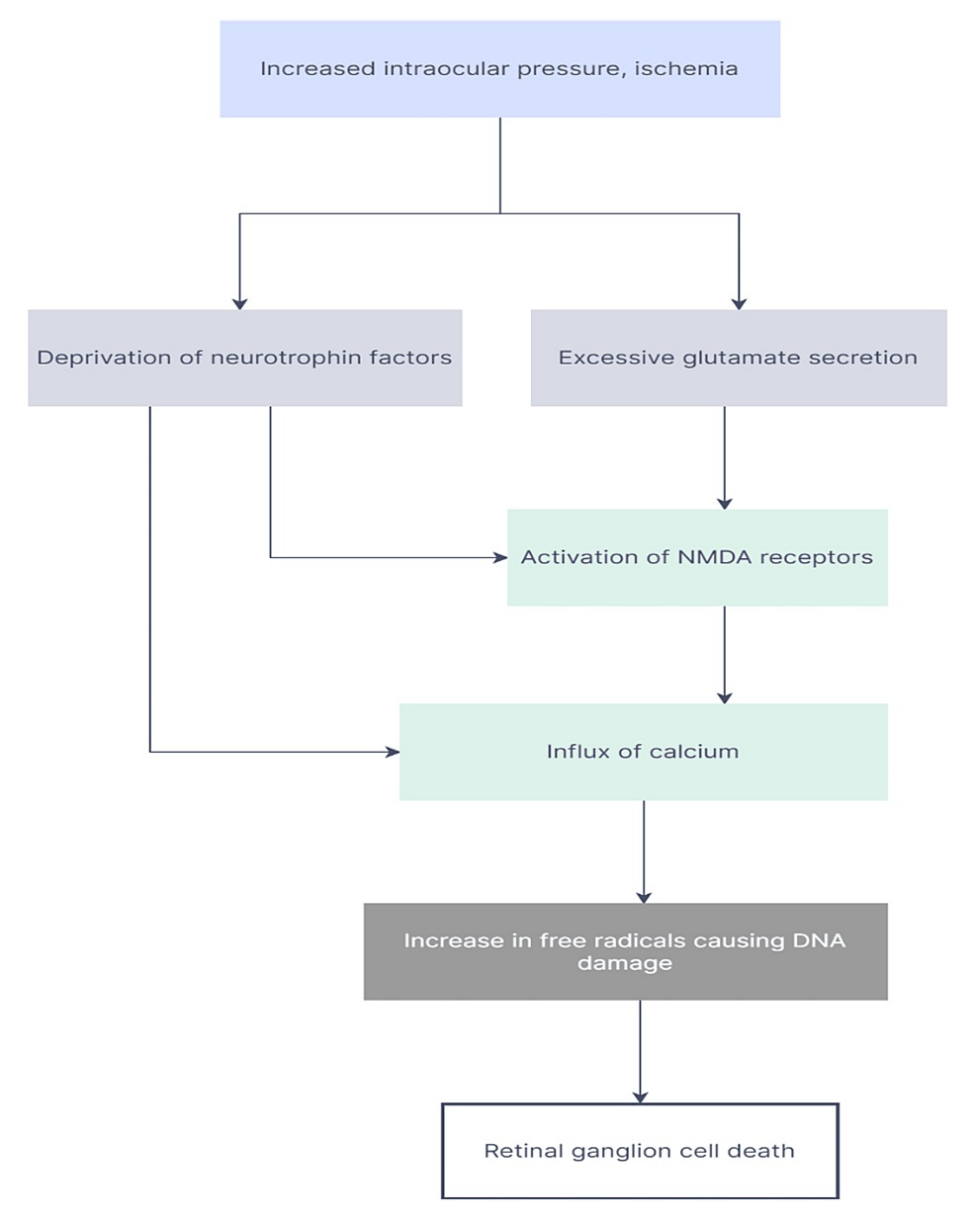
Different mechanisms of retinal ganglion cell death. NMDA: N-methyl-D-aspartic acid. This work is licensed and distributed by Creative Commons Attribution-NonCommercial-ShareAlike 3.0 License. Copyright © Journal of Ophthalmic and Vision Research [12].

Other than mechanical damage due to increased intraocular pressure, many mechanisms have been found that form the basis of retinal cell death, such as chronic ischemia, free radical formation, excitotoxicity, decreased neurotrophic factors, and malfunctioning axon transport (Figure 1). Chronic ischemia can occur, leading to decreased optic nerve perfusion, resulting in cell death. Free radicals or reactive oxygen species cause oxidation and excessive DNA damage to the cells leading to the destruction of the cells. Excitotoxicity is another phenomenon that can occur wherein the release of neurotransmitter glutamate causes excessive stimulation of N-methyl-D-aspartic acid receptors and consequently causes an influx of calcium and synthesis of nitric oxide that ultimately results in metabolic dysfunction and oxidative stress. Neurotrophic factors are growth factors essential for the growth and renewal of neuronal cells. Deprivation of these neurotrophic factors leads to progressive damage of retinal ganglion cells. Defective axon transport can occur due to mechanical stress or ischemia that causes a blockade of ineffective axonal communication and, therefore, damage to ganglion cells. All these mechanisms go hand in hand, and, on activation, ultimately lead to retinal ganglion cell death [11].

Neuroprotection in glaucoma

Because the restorative capability of neurons post-trauma or degeneration is limited, damage to neuronal cells can prove fatal as it affects their functionality. The extent of damage in glaucoma leading to the central nervous system has been repeatedly demonstrated in animal models, with a primary focus on monkeys due to significant anatomical and physiological similarities in the visual pathway of humans and primates. Overall, these findings point toward the knowledge that lowering intraocular pressure is not the only standalone factor, and may not be adequate in preventing glaucoma and delaying blindness. This forms the rationale for the current novel strategies, where neuroprotection can be considered an alternative for halting the progression of glaucoma. These strategies deal with molecular interventions that prevent neuronal damage by acting on factors at a cellular level that cause retinal cell death [13]. Neuroprotection is defined as the ability to regenerate or restore the neuronal cells that have endured degeneration or injury.

Reducing intraocular pressure as a whole contributes to delaying retinal cell death, and is indirectly seen as neuroprotective. However, neuroprotection in terms of glaucoma can be defined as any therapeutic intervention other than reduced intraocular pressure that aids in the prevention of retinal ganglion cell death [12].

Neuroprotection interventions in glaucoma include pharmacological as well as stem cell transplantation. Pharmacological therapeutic agents for neuroprotection include: Rho kinase inhibitors; glutamate antagonists like memantine and citicoline that prevent excitotoxicity; alpha two adrenergic agonists like brimonidine, calcium channel blockers, statins, and antioxidants against the free radical formation; neurotrophic factors; and nitric oxide synthase inhibitors. Cell transplantation includes stem cell transplants, nerve extracts, and Schwann cells as a means of treatment. Discussion of all these treatment modalities is beyond the scope of this review; hence, it will solely focus on Rho kinase inhibitors as a neuroprotective agent in glaucoma.

Rho kinase inhibitors

Rho kinase inhibitors are the latest addition to anti-glaucoma drugs and belong to the ocular hypotensive class. Their neuroprotective mechanism has been investigated recently, showing its prevalence in the regeneration of axons and promoting cell survival.

Rho Kinase Pathway

Rho kinase/guanine triphosphate (GTP)ase pathway acts as an essential regulator for cellular movement and cytoskeletal dynamics. Rho proteins are stimulated by guanine nucleotide exchange factors which are monitored by receptors present on the plasma membrane. On activation, ras homolog family member A (RhoA) gets bound to GTP, which subsequently activates Rho-associated protein kinase (ROCK). ROCK occurs as two isoforms, ROCK1 and ROCK2, which phosphoryla various substrates, mainly myosin light chain and myosin phosphatase substrate 1, LIM kinase 1 derived from LIMK1 gene, calponin, and ezrin-radixin-moesin proteins. This results in cellular adaptations like actin-myosin excitation-contraction coupling, cell adhesion, cell migration, extracellular matrix reorganization, and neurite retraction, among others [14].

Therapeutic Effects of Rho Kinase Inhibitors in the Treatment of Glaucoma

**Figure 2:**
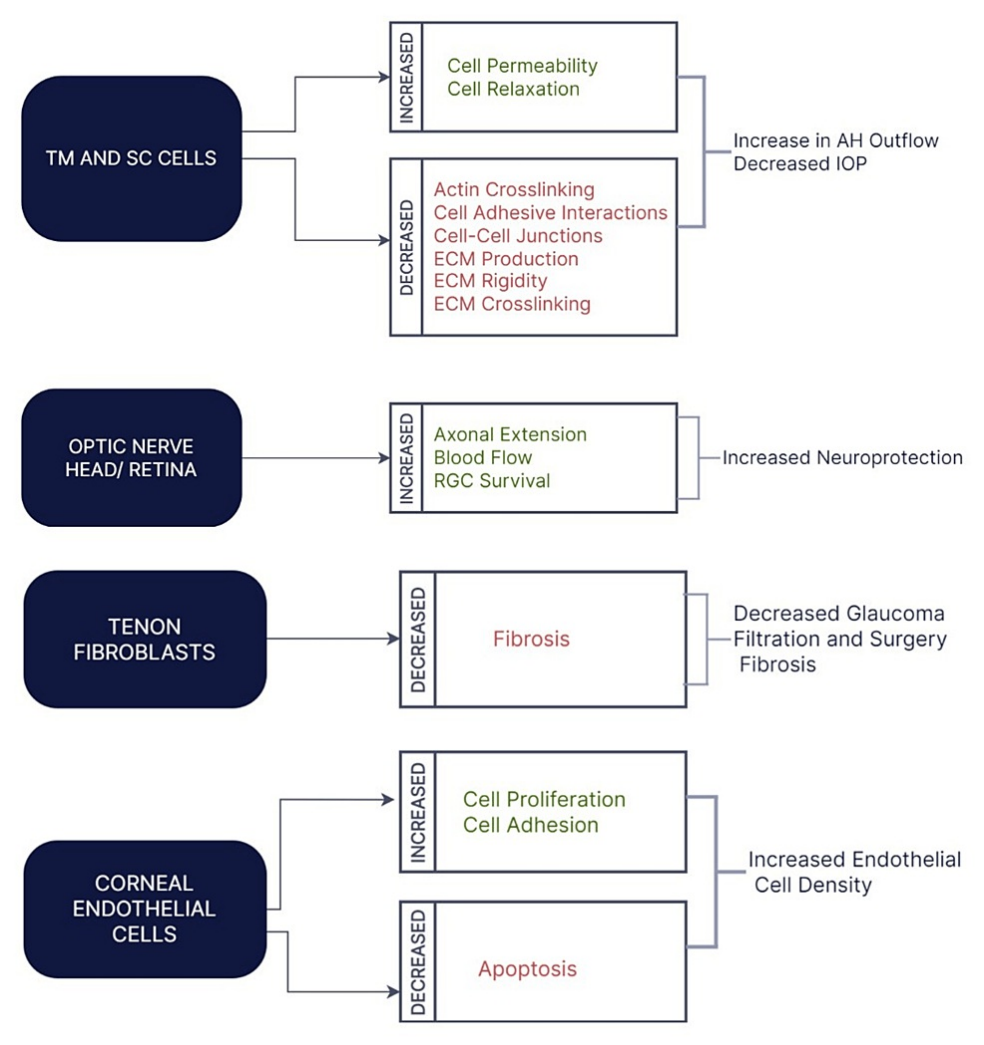
Therapeutic effects of Rho kinase inhibitors. TM: trabecular meshwork; SC: Schlemm's canal; IOP: Intraocular pressure; ECM: Extracellular matrix; RGC: Retinal ganglion cells. This work is published and licensed by Dove Medical Press Limited https://www.dovepress.com/terms.php. Copyright © 2021 Al-Humimat et al. [21].

Rho kinase inhibitors have shown multiple inhibitory effects that aid in the prevention and progression of glaucoma (Figure 2). Rho kinase inhibitors block the contraction of trabecular meshwork cells and cause an increase in the outflow of aqueous humor, thereby decreasing the intraocular pressure. A study conducted to demonstrate the inhibitory action of ROCK1 inhibitors on trabecular meshwork cytoskeleton and extracellular matrix production through an in vitro model revealed a reduction in intraocular pressure [15]. A clinical study showed that SNJ-1656, a ROCK inhibitor with intraocular-reducing properties, is safe for topical administration, as adverse systemic side effects can be avoided due to its confinement to a particular area of the body [16]. ROCK inhibitors are now used as the latest treatment modality in reducing intraocular pressure levels due to their success in clinical trials, notably ripasudil and netasurdil. Recent studies of netasurdil in combination with Latanoprost have shown additive effects in reducing intraocular pressure and are considered promising in glaucoma treatment [17].

When used topically in rabbit models, Rho kinase inhibitors AMA0526 and Y-27632 depict effects at different levels of the wound healing process. After glaucoma filtration surgery, there is a relatively drastic improvement in the outcome by decreasing scar formation and fibroproliferation by inactivation of conjunctival fibroblasts [18,19].

Rho kinase inhibitors have shown therapeutic effects on corneal endothelial regeneration and restoration of damaged endothelial cells. Ripasudil, a ROCK inhibitor, shows maintenance of corneal endothelial cell function and density and has a protective effect on corneal endothelium after ocular surgery; hence, this can be used therapeutically for the treatment of glaucoma [20].

Neuroprotective Activity of Rho Kinase Inhibitors

Rho kinase inhibitors have shown essential and radical effects in neurodegenerative diseases, with particular emphasis on their neuronal regeneration and cell survival properties. A combination of these factors has shown tremendous improvement in the therapeutic outlook for neuroprotection. Various studies have noted the significance of Rho kinase inhibitors as a treatment for neurodegenerative illnesses, although neurite growth has been best when seen at an embryonic or early developmental stage [22].

In one study, a ROCK2 inhibitor showed a specific therapeutic target against neurodegeneration, where the effects of ROCK2 were downregulated by using specific adeno-associated viral vectors. These were used in experiments on retinal ganglion cells of rats in vivo and in vitro, which led to significant neurite outgrowth and axonal sprouting and regeneration [14].

A particular study assessed the neurite outgrowth of ROCK inhibitors Y-27632, fasudil, and dimethyfasudil in vitro and in vivo and noted significant results. It was first experimented with in vitro using ntera-2 neurons on chondroitin sulfate proteoglycan (CSPG) substrates that act as an inhibitory agent in regenerative response by activating the ROCK pathway. All three ROCK inhibitors showed adequate neurite outgrowth despite the presence of CSPG substrates. In vivo testing conducted on the optic nerve crush model, ROCK inhibitors Y-27632 and dimethyfasudil were used, and both showed axonal regeneration. Y-27632 showed maximum growth when used at its most potent concentration, although dimethyfasudil showed maximum growth when regulated at midway concentrations [23].

Regeneration of neurons is mainly restricted due to pro-apoptotic factors inducing cell death post insult and inhibitory molecules preventing axonal elongation. Thus, interaction based on the inhibition of these two factors can be effective. The creation of a growth-promoting milieu with the help of ROCK inhibitor Y-27632 and ciliary neurotrophic factor has shown additive effects in facilitating retinal ganglion cell survival and neurite restoration compared to when used alone [24,25]. In the case of spinal cord injuries, prevention of axon regeneration is attributed to the growth inhibitory environment present in white matter. It was demonstrated that the inactivation of the Rho kinase pathway with a Rho kinase inhibitor Y27632 showed sufficient axon growth and can be used for recovery after spinal cord injury due to its neuroprotective ability [26].

Rho kinase inhibitor, Y-39983, is a potential drug candidate that can be used, and it was tested for treatment in glaucoma, where it showed increased vasodilation in the optic nerve of rabbits and had the potential for axonal regeneration of retinal neuronal cells seen in rats [27]. A new Rho inhibitor, K-115 or ripasudil, was administered as a treatment modality for glaucoma using an optic nerve crush model for the axon regenerative capacity. The retinal ganglion cell survival in the model was measured using a real-time polymerase chain reaction. K-115 showed significant neuroprotective ability and can be considered a new therapeutic drug for the treatment of glaucoma [28].

Limitations

The low regenerative capacity of adult neurons is a significant limitation, as overcoming the inhibitory environment for neuronal growth may not be sufficient to propagate neuroprotection [22]. Delay in treatment can prove to be ineffective in inducing neuronal regeneration in case of long-term injury. This is most likely due to the excessive scarring, despite the growth promotion produced by Rho inhibitors, as seen in a study where a Rho kinase inhibitor was administered four weeks post injury and showed no recovery [29]. As most of the Rho inhibitors are dose-dependent, their efficacy can be questioned due to their narrow therapeutic index. Low-dose administration can prove detrimental due to the inability to overcome inhibitory pathways, while high dosage can lead to undesirable adverse effects and intolerance [30]. The main limitation noted is that neuron regeneration is intricate and replicates the same inhibitory environment through substrates, so examining the functional efficacy of a drug can be challenging. Because more than one inhibitory mechanism is involved in preventing the regeneration of neurons in the brain, developing a drug that can overcome all these hurdles is still underway.

## Conclusions

Glaucoma is a degenerative disease that has been prevalent and poses a challenge in terms of treatment and early diagnosis. Treatment interventions have evolved considerably over the years and have been researched in detail. The use of rho kinase inhibitors as a treatment modality under the ocular hypotensive class has been established, but the use of rho kinase inhibitors beyond reducing intraocular pressure is still underway. The potential of neuroprotection offered by rho kinase inhibitors shows significant progress and should be explored further, as it is associated with axon regeneration and retinal ganglion cell survival. Although its neuroprotective abilities have limitations in accordance to the type and duration of injury, with further research it can be better understood and applied for therapeutic purposes.

The usage of neuroprotective medications for the treatment of glaucoma should be considered, as the addition of a neuroprotective agent in combination with ocular hypotensives can help in the prevention and progression of the disease and help prevent complications such as blindness on a larger scale.
